# Efficacy of multivitamin support following bariatric surgery in patients with obesity: a prospective observational study

**DOI:** 10.1007/s40519-024-01655-7

**Published:** 2024-05-07

**Authors:** Alessio Basolo, Susanna Bechi Genzano, Jacopo Vitti, Guido Salvetti, Donatella Gilio, Giovanni Ceccarini, Giovanna Scartabelli, Chita Lippi, Rosario Bellini, Rudi Mancini, Simone D’Imporzano, Carlo Moretto, Valentina Angeli, Daniela Troiani, Paola Fierabracci, Roberta Jaccheri, Alba Calderone, Anello M. Poma, Luca Chiovato, Giorgio Saponati, Ferruccio Santini

**Affiliations:** 1 Obesity and Lipodystrophy Center, Endocrinology Unit, University Hospital of Pisa, 56124 Pisa, Italy; 2 Bariatric Surgery Unit, University Hospital of Pisa, 56124 Pisa, Italy; 3 Department of Surgical, Medical, Molecular Pathology and Critical Care Medicine, University of Pisa, 56100, Pisa, Italy; 4 Istituti Clinici Scientifici Maugeri IRCCS, 27100 Pavia, PV Italy; 5 ISPharm CRO, 55100 Lucca, Italy; 6 University of Pisa, Pisa, Italy

**Keywords:** Obesity, Bariatric surgery, Micronutrients, Multivitamin support

## Abstract

**Purpose:**

Bariatric surgery (BS), an effective treatment for severe obesity and its comorbidities, may result in micronutrient and vitamin deficiencies. This monocentric prospective observational study aimed at evaluating the efficacy of a specifically designed vitamin/mineral formula (Bariatrifast, BIOITALIA S.r.l., Italy) for preventing and treating micronutrient deficiencies in patients submitted to BS.

**Methods:**

Twenty patients with severe obesity (mean weight and BMI: 123.5 kg (range 88–174) and 43.3 kg/m^2^ (range 37–54) respectively) underwent BS (10 vertical sleeve gastrectomy VSG, 10 Roux-en-Y gastric bypass, RYGB). The mean age was 49.9 years (range 27–68). After a presurgical visit (V0), follow-up visits were performed at 1, 3, 6 and 12 months after surgery (V1–V4). Recorded data included weight, height and BMI. A complete blood count, measurement of ferritin, folic acid, vitamin B12, ionized calcium, 25 OH vitamin D, parathyroid hormone (PTH) were obtained. Following BS, patients started the daily oral multivitamin and mineral supplement.

**Results:**

All patients achieved a significant weight loss (mean − 34.7 ± 11.8 kg). No deficiencies of various vitamins/micronutrients were detected during the entire study period. The serum concentrations of vitamin B12, 25-OH Vitamin D and folic acid increased over the follow-up period compared with V0 (mean increase 243 ng/L, 23 µg /L, 8 µg/L, respectively). Compared to RYGB, patients who underwent sleeve gastrectomy showed higher levels of 25-OH vitamin D at V2, V3 and V4 (all *p* < 0.05), and higher levels of Vitamin B12 and folic acid at V4 (*p* < 0.05 and *p* < 0.005, respectively). No adverse events were reported.

**Conclusion:**

Following VSG or RYGB, Bariatrifast administration was associated with normal values of essential micronutrients, and it was well-tolerated without evidence of gastrointestinal side effects.

*Clinical Trial Registration* ClinicalTrials.gov, identifiers NCT06152965

**Supplementary Information:**

The online version contains supplementary material available at 10.1007/s40519-024-01655-7.

## Introduction

Obesity is a chronic condition characterized by the accumulation of excess adipose tissue, posing a significant public health concern [1, 2]. According to the World Health Organization, approximately 13% of adults worldwide have a body mass index (BMI) that, being equal to or exceeding 30 kg/m^2^, indicates an obesity condition [3]. The main goal of obesity treatment is weight loss that may be achieved by lifestyle intervention as first approach. Pharmacological treatment and bariatric surgery (BS) may be considered in adjunct to lifestyle intervention if the latter was not sufficient [4]. Pharmacotherapy can assist affected patients to lose weight and reduce health risks associated with obesity [5]. However, BS stands up as the most effective treatment for severe obesity [4]. Thus, BS is increasingly performed in patients with severe obesity, as assessed by BMI ≥ 40 kg/m^2^ or even lower when associated with specific comorbidities [6, 7]. Vertical sleeve gastrectomy (VSG) and Roux-en-Y gastric bypass (RYGB) are the most frequently used surgical procedures [8]. Although BS effectively reduces body weight, improves metabolic alterations, and enhances overall health [9], it may also lead to micronutrients deficiency [6, 10, 11]. The mechanisms involved in the development of vitamins and mineral deficiency include mainly decreased caloric intake and possibly diminished nutrient absorption due to reduced gastric pouch size (in RYGB) and rearrangement of the digestive tract leading to pH alterations (in both VSG and RYGB) [12]. According to recent guidelines [11, 13], it is recommended to implement systematic vitamin and micronutrients’ supplementation following different surgical procedures, with the aim to ensure sufficient nutritional intake and decrease the risk of deficiencies [14–16]. Several Food (supplements) for Special Medical Purposes (FSMP) are available to improve the nutritional intervention.

This prospective observational study aimed at evaluating the effectiveness and safety of a specific FSMP (Bariatrifast, BIOITALIA S.r.l., Italy) in maintaining an adequate vitamin and micronutrient intake in patients with obesity who underwent VSG or RYGB.

## Materials and methods

This observational, prospective study included 20 consecutive patients with severe obesity who underwent BS (11 males, 9 females, all aged ≥ 18 years, with a BMI ≥ 40 kg/m^2^ or ≥ 35 kg/m^2^ with at least one obesity-related comorbidity). In our population, 10 patients had hypertension, 5 type 2 diabetes, 2 non-alcoholic liver disease, 7 gastroesophageal reflux disease, 7 dyslipidemia and 2 obstructive sleep apnea. Ten of them underwent VSG and 10 RYGB. Patients were recruited at the Obesity and Lipodystrophy Center, University Hospital of Pisa from April 2021 to February 2023. Previous BS was an exclusion criterion. The choice of the BS procedure was done by a multidisciplinary team based on medical history and obesity-related comorbidities.

Starting from the third day after surgery, a daily tablet of Bariatrifast (supplied free of charge by BIOITALIA S.r.l., Italy) was administered. Each pill contained 65 mg of iron,  175 μg of 25-OH Vitamin D (equivalent to 7000 IU), and several other micronutrients, including vitamins (A, B1, B2, B3, B5, B6, B8, B9, B12, C, E, K), zinc, copper, and selenium (Table 1). The study protocol included a presurgical visit (V0), and 4 follow-up visits at 1 (V1), 3 (V2), 6 (V3), and 12 (V4) months after BS. The follow-up period between V0 and V4 was 373 ± 6 days (mean ± SD).

**Table 1:** Composition of micronutrients and vitamins in each tablet of Bariatrifast

Composition	
Magnesium (mg)	56.3
Iron (mg)	65.0
Zinc (mg)	10.0
Copper (mg)	1.0
Selenium (µg)	55.0
C Vitamin (mg)	120.0
E Vitamin (mg)	100.0
Thiamine (mg)	10.0
Riboflavin (mg)	1.3
B6 Vitamin (mg)	1.5
Pantothenic acid (mg)	10.0
Niacin (mg)	10.0
A Vitamin (µg)	1200.0
Folic acid (µg)	400.0
Biotin (µg)	50.0
B12 Vitamin (µg)	500.0
Cholecalciferol (D Vitamin) (µg)	175.0*
K Vitamin (µg)	150.0

*Equal to 7.000 I.U

Body weight, blood pressure and heart rate were recorded at each visit. Blood tests were also performed and included a complete blood count, measurement of ferritin, ionized calcium, 25-OH Vitamin D, PTH, vitamin B12, folic acid. Blood was drawn after an overnight fast. A digital electronic scale was used to assess body weight, in light clothing. Standing height, without shoes, was measured (to the nearest 1 cm) using a stadiometer. Body mass index (BMI) was calculated as the weight in kilograms divided by the square of the height in meters. Classification of overweight and obesity was performed according to conventional definitions [17]. The adherence to the treatment was evaluated for each patient at the end of the follow-up period and it was expressed as a percentage ratio between the number of tablet taken and those supposed to be taken in relation to the treatment period.

The study was approved by the local Ethical Committee (CEAVNO—Comitato Etico Area Vasta Nord Ovest Regione Toscana) and all patients gave their written informed consent.

### Statistical analysis

Data were analyzed according to the intention-to treat procedure. Normally distributed variables were expressed as arithmetic mean ± standard deviation (SD). *p* values < 0.05 were considered significant. Changes from baseline (pre-surgery) parameters were evaluated using the Student’s paired *t* test or non-parametric tests, as appropriate. Weight change was calculated in both VSG and RYGB groups, and difference between the 2 groups was assessed by Student’s unpaired *t* test. Two-way ANOVA for repeated measures was used to explore changes in micronutrients/vitamins serum levels over and according to surgical procedures. Post-hoc pairwise *t* test with Bonferroni correction was used to highlight differences among the two groups at different time points. Differences in safety, tolerability and adherence between the groups were compared by Chi-squared tests.

## Results

**Fig. 1:**
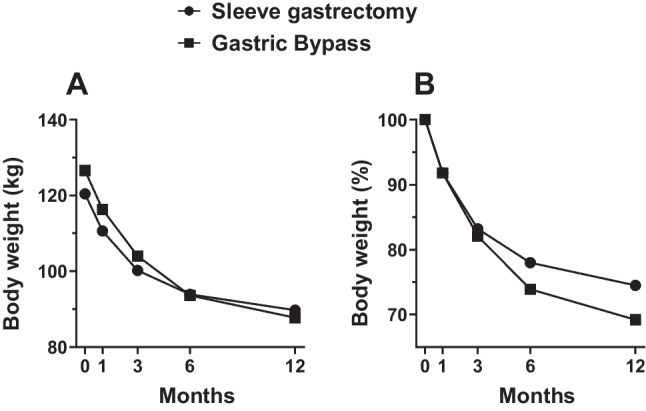
**A** Change in body weight (mean values at each visit) over 12 months in the VSG and RYGB groups. Time 0 corresponds to body weight before BS (V0). **B** Percent weight loss trajectory (mean values) in the VSG versus RYGB groups over 12 months

The anthropometric characteristics of the study population are reported in Table 2. At the time of the presurgical visit, no differences in age, body weight and BMI were observed between RYGB and VSG. Prior to BS, 25-OH Vitamin D deficiency (< 25 µg/L) and folate deficiency (< 3 µg/L) were observed in thirteen patients (65%) and 2 patients (10%), respectively. No abnormal values were observed in the other parameters. As expected, BS induced significant weight loss in all patients from V0 to V4 (Δ =  − 34.7 ± 11.8 kg, ranging from − 57 to − 13). Patients who underwent the RYGB intervention showed a higher percentage of weight loss (− 31 ± 7%) compared with the VSG group (− 25 ± 8%) (Fig. 1), although not statistically significant.

**Table 2:** Anthropometric measures of the study population

	Whole group *n* = 20	RYGB *n* = 10	VSG *N* = 10	*p* value
Age (years)	49.9 (27–68)	46.4 (27–68)	53.4 (42–62)	0.1
Body weight (kg)	123.5 (88–174)	126.0 (88–146)	120.4 (91–174)	0.5
BMI (kg/m^2^)	43.3 (37–54)	41.4 (37–45)	45.1 (36–54)	0.09

Data are presented as mean (minimum–maximum)

*BMI* body mass index

**Fig. 2:**
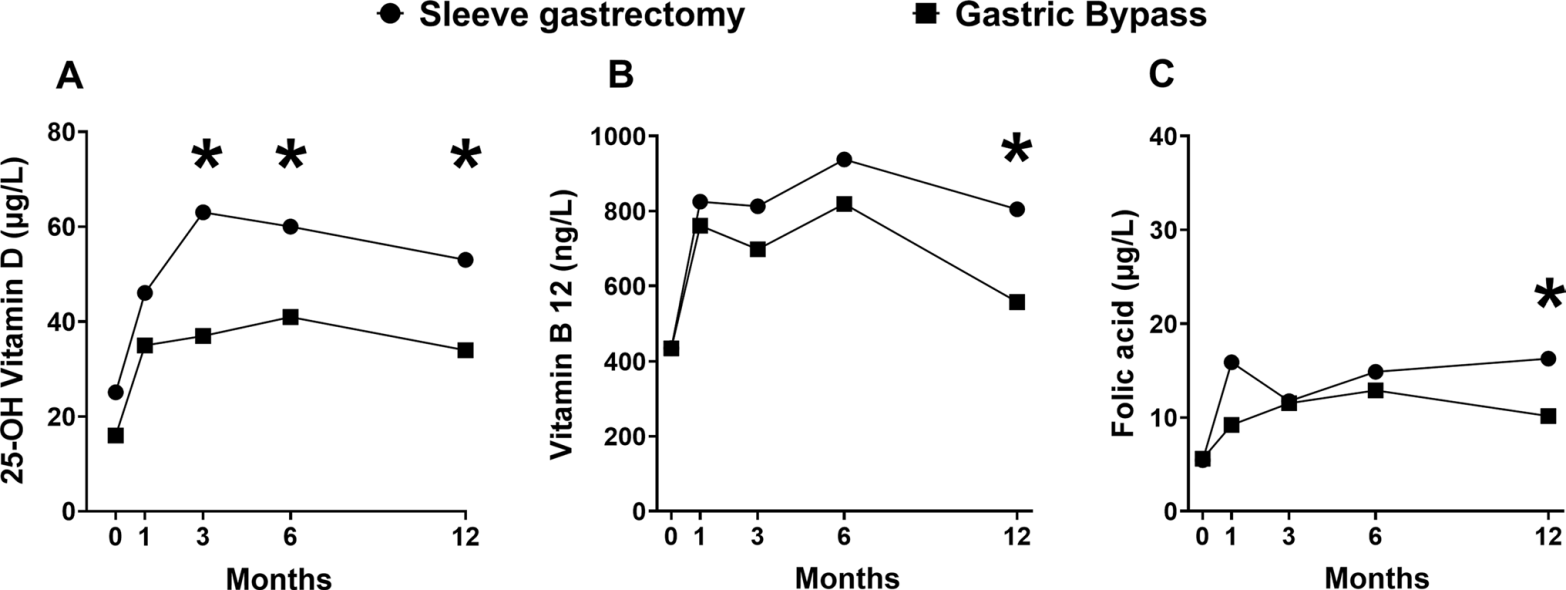
Changes in 25-OH Vitamin D (**A**), vitamin B12 (**B**) and folic acid (**C**) concentrations from baseline over 12 months in the VSG and RYGB. The asterisks represent the significant difference in vitamin levels between groups at each time point by post-hoc pairwise *t* test with Bonferroni correction (*p < 0.05)

Serum concentrations of vitamin B12, 25-OH Vitamin D and folic acid increased over the follow-up period compared with V0 (mean increase at V4 243 ng/L, 23 mcg/L and 8 µg/L, respectively). Using two-way ANOVA for repeated measures, serum levels of 25-OH vitamin D increased over time (*p* < 0.001) with a significant effect of surgical procedures (*p* < 0.001). Post-hoc pairwise *t* test with Bonferroni correction showed higher levels of 25-OH vitamin D in patients who underwent sleeve gastrectomy at V2, V3 and V4 (all *p* < 0.05, Fig. 2A). Vitamin B12 increased over time (*p* < 0.001), and post-hoc pairwise *t* test with Bonferroni correction showed higher levels of Vitamin B12 in patients who underwent sleeve gastrectomy at V4 (*p* < 0.05, Fig. 2B). Folic acid increased over time (*p* < 0.001) with a significant effect of surgical procedures (*p* < 0.05). Post-hoc pairwise *t*-test with Bonferroni correction showed higher levels of folic acid in patients who underwent sleeve gastrectomy at V4 (*p* < 0.005, Fig. 2C). Slightly higher values of PTH at V2 and V3 (*p* < 0.05) were observed after RYGB.

In 7 patients who achieved serum levels of Vit. B12 > 1.000 ng/L at V2 (1 patient with RYGB) and at V3 (4 with VSG and 2 with RYGB), Bariatrifast was replaced by another formula containing lower amounts of vitamins and micronutrients (Bariatric, BIOITALIA, Italy, Table 3) with consequent decline of Vit. B12 values below 1000 ng/L. In the 7 patients who achieved serum levels of Vitamin B12 > 1.000 ng/L, the reduction in body weight (− 39.3 ± 12.8 kg, ranging from − 54 to − 17) was not significantly different from that observed in the other patients (− 32.3 ± 10.9 kg, ranging from − 57 to − 13).

**Table 3:** Composition of micronutrients and vitamins in each tablet of Bariatric

Composition	
Magnesium (mg)	188
Iron (mg)	30
Zinc (mg)	10.0
Selenium (µg)	55.0
C Vitamin (mg)	120.0
E Vitamin (mg)	12.0
Thiamine (mg)	10.0
Riboflavin (mg)	2.1
B6 Vitamin (mg)	2.1
Pantothenic acid (mg)	9.0
Niacin (mg)	24.0
A Vitamin (µg)	800.0
Folic acid (µg)	400.0
B12 Vitamin (µg)	33.0
Cholecalciferol (D Vitamin) (µg)	25.0*
K Vitamin (µg)	38.0

^*^Equal to 1000 I.U

No significant changes in serum levels of blood counts, ferritin, and ionized calcium were observed (Table 4). In all patients, no deficiency nor subnormal level of various blood parameters was observed during FSMP administration. No adverse events were reported. Good adherence to oral supplementation was observed (median ratio between the number of tablets taken and tablets scheduled = 97.6% (range 77–100).

**Table 4:** Micronutrients and vitamin measures over the follow-up period stratified by surgical procedure

	Sleeve gastrectomy					Gastric bypass				
	V0	V1	V2	V3	V4	V0	V1	V2	V3	V4
Erythrocytes (× 10^3^/µL)	4971 (457)	4898 (485)	4923 (432)	4858 (556)	4799 (485)	4905 (404)	4899 (358)	4836 (406)	4716 (469)	4758 (432)
Hemoglobin (g/dL)	14.4 (1.3)	14.2 (1.4)	14.5 (1.1)	14.5 (1.4)	14.4 (1.3)	14.4 (1.4)	14.4 (1.3)	14.3 (1.3)	14.3 (1.5)	14.4 (1.6)
Leukocytes (/µL)	8279 (2216)	6119 (1080)	7508 (1170)	7346 (1184)	7427 (1200)	7134 (1313)	7531 (3211)	7641 (1291)	7708 (1116)	7271 (1213)
Platelets (× 10^3^/µL)	273.5 (59)	257.1 (61)	250.9 (48)	256.4 (46)	234.3 (34)	246.0 (86.9)	257.5 (75.9)	250.9 (63.6)	249.7 (69.7)	259.6 (88.3)
PTH (ng/L)	27.3 (5.8)	25.5 (3.9)	26.6* (8.1)	27.5* (7.7)	28.1 (6.9)	34.9 (14.7)	37.2 (16.9)	39.6 (12.8)	37.6 (7.4)	36.4 (11.9)
Folic acid (µg/L)	5.5 (4.1)	15.9 (11.0)	11.8 (4.5)	14.9 (4.3)	16.3* (5.5)	5.6 (3.2)	9.2 (4.5)	11.5 (5.0)	12.9 (4.5)	10.2 (4.3)
Ionized calcium (mmol/L)	1.23 (0.02)	1.24 (0.05)	1.20 (0.09)	1.21 (0.09)	1.23 (0.05)	1.25 (0.04)	1.23 (0.07)	1.24 (0.03)	1.23 (0.04)	1.23 (0.04)
25-OH Vitamin D (µg/L)	25.1 (14.3)	46.1 (8.7)	62.6* (8.7)	59.9* (10.8)	53.0* (13.4)	16.4 (8.4)	35.0 (10.3)	37.4 (6.2)	41.8 (14.5)	34.6 (9.0)
Ferritin (µg/L)	173.0 (197)	273.6 (222.6)	169.4 (115.5)	137.5 (73.5)	149.7 (104.3)	200.8 (106.4)	169.0 (115.9)	157.0 (61.8)	141.0 (62.9)	127.8 (67.1)
Vitamin B12 (ng/L)	434.9 (186.8)	825.0 (322.8)	812.5 (264.8)	937.4 (281.2)	804.5* (172.6)	434.7 (137.6)	760.9 (327.4)	697.8 (447.2)	819.0 (549.0)	557.4 (158.0)

Each parameter in the table is expressed as mean (standard deviation)

The asterisks represent the significant difference between groups at each time point by post-hoc pairwise *t* test with Bonferroni correction (*p < 0.05)

## Discussion

The current study demonstrates that a brand name vitamin/mineral formula (Bariatrifast) was associated with normal values of essential micronutrients during a 1-year follow-up period after VSG or RYGB. Good adherence and no side effects were observed.

BS can induce nutritional deficits that may exacerbate preexisting micronutrients deficiencies [18–21], which result from the poor nutritional quality of the diet, e.g. lack of fruits and vegetables (rich sources of vitamins and minerals), and from specific factors unique to patients with obesity [18, 22]. The prevalence of 25-OH vitamin D deficiency was reported to be 35% higher in individuals with obesity compared with normal weight subjects [23, 24]. Iron deficiency and low hemoglobin levels were reported to occur from 0 to 47% in individuals scheduled for BS [21, 25–27]. Folic acid deficiency rates before BS varied from 0 to 23% [18, 20, 27, 28], while vitamin B12 deficiency was reported to range from 0 to 23% [27, 29].

Starting from preexisting nutritional deficits, BS can make the situation worse. Iron deficiency is highly prevalent after VSG and RYGB, affecting approximately 33% of patients [30, 31], mainly in menstruating women [11]. This condition may be due to the reduced intake of iron-rich foods in the initial months after BS [32]. Current recommendations advise a daily intake of 18 mg of iron in men and non-anemic patients, and of 45–60 mg in menstruating women [11, 33]. Our results show that, by supplementing 65 mg of iron per day, the serum levels of iron were adequate during the whole follow-up period.

Vitamin B12 deficiency is a well-known complication of BS, and it is mainly due to reduced intake [11, 33]. Vitamin B12 undergoes degradation in the gastric acid environment. Consequently, cells in the stomach release intrinsic factor, which binds to vitamin B12. This process takes place in the duodenum, with actual absorption occurring in the ileum [34]. When the intestinal tract is either bypassed or altered (e.g. after gastric bypass or duodenal switch), a reduction in vitamin B12 absorption may occur [35]. Some studies reveal that after RYGB, vitamin B12 deficiency is more prevalent than after VSG [36, 37]. Recommended vitamin B12 supplementation varies based on the administration method: either a daily oral intake of 350–1000 μg or a monthly intramuscular injection of 1000 μg is advised, regardless of the surgery type [33, 35]. The results of the present study indicate that the oral administration of 500 μg of vitamin B12 produces an increase in serum vitamin B12 concentrations that, throughout the study, were found to be in the normal range in the majority of investigated patients. As stated by the NIH office for dietary supplements, the Food and Nutrition Board of the Institute of Medicine does not establish a upper intake level for Vitamin B12 [38], due to its low potential toxicity even at large doses. Vitamin B12 is generally considered to be safe because the body does not store excess amounts. Despite none of the 7 patients who achieved serum levels of Vitamin B12 > 1.000 ng/L complained of any side effect, we decided to replace Bariatrifast with an oral formula containing a lower amount of this vitamin.

Folic acid deficiency is also observed after BS due to inadequate intake [11, 36, 39]. Generally, patients are recommended a daily 400–800 μg dose, while reproductive-age women might require 800–1000 μg daily [11]. We observed that the administration of 400 µg prevented folic acid deficiency, while leading to a progressive increase in its levels, though remaining within the normal range over the follow-up period.

Anemia frequently occurs after BS. A recent meta-analysis demonstrated a similar prevalence of anemia in obese patients submitted to either RYGB or VSG [36]. In our study population, hematological parameters, including hemoglobin levels, remained within the normal range throughout the treatment/observation period, and no case of anemia was recorded. This successful outcome can be attributed to the normalization of iron, vitamin B12 and folic acid.

Independently of the type of BS [40], 25-OH Vitamin D deficiency occurs in 25–73% of operated patients, and it may lead to decreased bone mineral density and worsened bone turnover [41, 42]. Some studies show a consistent increase in fracture risk after RYGB compared with other procedures [43–45], but randomized controlled trials do not report a higher risk of fractures in BS-treated patients compared with controls [46–48]. In patients with obesity who followed a dietary intervention promoting important weight loss, an increase in 25-OH vitamin D has been reported [49]. Since Vitamin D is stored in adipose tissue, its elevation may be due to increased release consequent to loss in fat mass.

Current guidelines recommend administering at least a 2000 IU daily supplementation and periodically monitoring 25-OH Vitamin D levels [24]. In our study, the administration of 175 µg (7000 UI/day) of 25-OH Vitamin D increased the serum level of this vitamin, which were found to be ≥ 25 µg/L in all subjects, even in those starting with a presurgical level < 20 ng/ml. Low 25-OH Vitamin D levels can reduce intestinal calcium absorption. Consequently, the parathyroid glands may increase the production of PTH to balance serum calcium levels, potentially resulting in secondary hyperparathyroidism. Our results showed normal and stable serum values of PTH and ionized calcium throughout the course of the study.

Vitamin B1 (thiamine) deficiency can occur after BS due to inadequate intake or persistent vomiting [11, 50]. After BS, the prevalence of thiamine deficiency is estimated at 18% of operated patients [51]. Vitamin B1 deficiency can have consequences on the cardiovascular, nervous, and immune systems, resulting in conditions such as wet beriberi, dry beriberi, or Wernicke–Korsakoff syndrome [50, 52]. The recommended daily intake of thiamin is 1.2 mg per day in men and 1.1 mg per day in women [13, 53]. However, the American Society for Parenteral and Enteral Nutrition suggests a thiamine intake ranging from 1.2 mg to a maximum of 10 mg per day [13, 30, 54]. In our study cohort, we did not observe any clinical sign of vitamin B1 deficiency by administering 10 mg/day of this vitamin.

The post-surgical management of patients is critical because of the BS-induced decrease of nutrients intake and/or absorption [55, 56]. To prevent micronutrient deficiencies, the early administration of targeted food supplements after BS is crucial [21]. However, ensuring patients’ compliance with multiple supplements can be challenging and it may lead to a high incidence of unsuccessful outcomes, which may increase the sanitation costs due to a rise in emergency admissions, hospitalizations, and treatment expenses [57]. Simplifying the regimen into a single product may enhance adherence and minimize the risk of nutritional deficits [58].

Our study has some limitations. After BS, there is a potential risk to develop deficiencies of additional vitamins (A, K, E, B6) and micronutrients (copper, zinc, magnesium) [11, 20, 33]. The recommended doses of these micronutrients are included in Bariatrifast, although no specific measures were obtained to verify their efficacy. The relatively small sample size, lack of a control group undergoing specific dietary regimen, reliance on self-reported compliance with supplement regimens and the short follow-up period might limit the study's ability to draw complete conclusions.

## Conclusion

In the current study, the administration of an FSMP (Bariatrifast) was associated with normal values of vitamins and minerals in patients with obesity who underwent BS. A satisfactory adherence to Bariatrifast treatment and its safety were recorded.

### Supplementary Information

Below is the link to the electronic supplementary material.

Supplementary file1 (DOCX 31 KB)

## Data Availability

Some or all datasets generated during and/or analyzed during the current study are not publicly available but are available from the corresponding author on reasonable request.

## References

[1] Janssen F, Bardoutsos A, Vidra N (2020) Obesity prevalence in the long-term future in 18 European countries and in the USA. Obes Facts 13(5):514–527 33075798 10.1159/000511023 PMC7670332

[2] Blüher M (2019) Obesity: global epidemiology and pathogenesis. Nat Rev Endocrinol 15(5):288–298 30814686 10.1038/s41574-019-0176-8

[3] World Health Organization. Obesity and overweight fact sheet; 2016. https://www.who.int/news-room/fact-sheets/detail/obesity-and-overweight. Accessed 18 Sept 2021.

[4] Yumuk V, Tsigos C, Fried M, Schindler K, Busetto L, Micic D, Toplak H (2015) European guidelines for obesity management in adults. Obes Facts 8(6):402–424 26641646 10.1159/000442721 PMC5644856

[5] Toplak H, Woodward E, Yumuk V, Oppert JM, Halford JC, Frühbeck G (2015) 2014 EASO position statement on the use of anti-obesity drugs. Obes Facts 8(3):166–174 25968960 10.1159/000430801 PMC5644876

[6] Di Lorenzo N, Antoniou SA, Batterham RL, Busetto L, Godoroja D, Iossa A, Carrano FM, Agresta F, Alarçon I, Azran C, Bouvy N, Balaguè Ponz C, Buza M, Copaescu C, De Luca M, Dicker D, Di Vincenzo A, Felsenreich DM, Francis NK, Fried M, Gonzalo Prats B, Goitein D, Halford JCG, Herlesova J, Kalogridaki M, Ket H, Morales-Conde S, Piatto G, Prager G, Pruijssers S, Pucci A, Rayman S, Romano E, Sanchez-Cordero S, Vilallonga R, Silecchia G (2020) Clinical practice guidelines of the European Association for Endoscopic Surgery (EAES) on bariatric surgery: update 2020 endorsed by IFSO-EC. EASO and ESPCOP Surg Endosc 34(6):2332–2358 32328827 10.1007/s00464-020-07555-y PMC7214495

[7] Gildea A, Shukla S, Parretti H, Khan O (2023) Referral criteria and assessment for bariatric surgery: summary of updated NICE guidance. BMJ 382:1880 37643791 10.1136/bmj.p1880

[8] Angrisani L, Santonicola A, Iovino P, Vitiello A, Zundel N, Buchwald H, Scopinaro N (2017) Bariatric surgery and endoluminal procedures: IFSO worldwide survey 2014. Obes Surg 27(9):2279–2289 28405878 10.1007/s11695-017-2666-x PMC5562777

[9] Adams TD, Davidson LE, Litwin SE, Kim J, Kolotkin RL, Nanjee MN, Gutierrez JM, Frogley SJ, Ibele AR, Brinton EA, Hopkins PN, McKinlay R, Simper SC, Hunt SC (2017) Weight and metabolic outcomes 12 years after gastric bypass. N Engl J Med 377(12):1143–1155 28930514 10.1056/NEJMoa1700459 PMC5737957

[10] Patel JJ, Mundi MS, Hurt RT, Wolfe B, Martindale RG (2017) Micronutrient deficiencies after bariatric surgery: an emphasis on vitamins and trace minerals [formula: see text]. Nutr Clin Pract 32(4):471–480 28609642 10.1177/0884533617712226

[11] Busetto L, Dicker D, Azran C, Batterham RL, Farpour-Lambert N, Fried M, Hjelmesæth J, Kinzl J, Leitner DR, Makaronidis JM, Schindler K, Toplak H, Yumuk V (2017) Practical recommendations of the obesity management task force of the European Association for the study of obesity for the post-bariatric surgery medical management. Obes Facts 10(6):597–632 29207379 10.1159/000481825 PMC5836195

[12] Barazzoni R, Gortan CG (2020) Double burden of malnutrition in persons with obesity. Rev Endocr Metab Disord 21(3):307–313 32766943 10.1007/s11154-020-09578-1 PMC7455581

[13] Tabesh MR, Maleklou F, Ejtehadi F, Alizadeh Z (2019) Nutrition, physical activity, and prescription of supplements in pre- and post-bariatric surgery patients: a practical guideline. Obes Surg 29(10):3385–3400 31367987 10.1007/s11695-019-04112-y

[14] Coupaye M, Rivière P, Breuil MC, Castel B, Bogard C, Dupré T, Flamant M, Msika S, Ledoux S (2014) Comparison of nutritional status during the first year after sleeve gastrectomy and Roux-en-Y gastric bypass. Obes Surg 24(2):276–283 24122661 10.1007/s11695-013-1089-6

[15] Verger EO, Aron-Wisnewsky J, Dao MC, Kayser BD, Oppert JM, Bouillot JL, Torcivia A, Clément K (2016) Micronutrient and protein deficiencies after gastric bypass and sleeve gastrectomy: a 1-year follow-up. Obes Surg 26(4):785–796 26205215 10.1007/s11695-015-1803-7

[16] Aron-Wisnewsky J, Verger EO, Bounaix C, Dao MC, Oppert JM, Bouillot JL, Chevallier JM, Clément K (2016) Nutritional and protein deficiencies in the short term following both gastric bypass and gastric banding. PLoS ONE 11(2):e0149588 26891123 10.1371/journal.pone.0149588 PMC4758752

[17] Bray GA, Heisel WE, Afshin A, Jensen MD, Dietz WH, Long M, Kushner RF, Daniels SR, Wadden TA, Tsai AG, Hu FB, Jakicic JM, Ryan DH, Wolfe BM, Inge TH (2018) The science of obesity management: an endocrine society scientific statement. Endocr Rev 39(2):79–132 29518206 10.1210/er.2017-00253 PMC5888222

[18] Krzizek EC, Brix JM, Herz CT, Kopp HP, Schernthaner GH, Schernthaner G, Ludvik B (2018) Prevalence of micronutrient deficiency in patients with morbid obesity before bariatric surgery. Obes Surg 28(3):643–648 28849358 10.1007/s11695-017-2902-4

[19] Kobylińska M, Antosik K, Decyk A, Kurowska K (2022) Malnutrition in obesity: is it possible? Obes Facts 15(1):19–25 34749356 10.1159/000519503 PMC8820192

[20] O’Kane M, Parretti HM, Pinkney J, Welbourn R, Hughes CA, Mok J, Walker N, Thomas D, Devin J, Coulman KD, Pinnock G, Batterham RL, Mahawar KK, Sharma M, Blakemore AI, McMillan I, Barth JH (2020) British Obesity and Metabolic Surgery Society Guidelines on perioperative and postoperative biochemical monitoring and micronutrient replacement for patients undergoing bariatric surgery-2020 update. Obes Rev 21(11):e13087 32743907 10.1111/obr.13087 PMC7583474

[21] Aguas-Ayesa M, Yárnoz-Esquíroz P, Olazarán L, Gómez-Ambrosi J, Frühbeck G (2023) Precision nutrition in the context of bariatric surgery. Rev Endocr Metab Disord. 10.1007/s11154-023-09794-5:1-13 36928810 10.1007/s11154-023-09794-5:1-13 PMC10020075

[22] Argyrakopoulou G, Konstantinidou SK, Dalamaga M, Kokkinos A (2022) Nutritional deficiencies before and after bariatric surgery: prevention and treatment. Curr Nutr Rep 11(2):95–101 35174473 10.1007/s13668-022-00400-9

[23] Pereira-Santos M, Costa PR, Assis AM, Santos CA, Santos DB (2015) Obesity and vitamin D deficiency: a systematic review and meta-analysis. Obes Rev 16(4):341–349 25688659 10.1111/obr.12239

[24] Giustina A, di Filippo L, Facciorusso A, Adler RA, Binkley N, Bollerslev J, Bouillon R, Casanueva FF, Cavestro GM, Chakhtoura M, Conte C, Donini LM, Ebeling PR, Fassio A, Frara S, Gagnon C, Latella G, Marcocci C, Mechanick JI, Minisola S, Rizzoli R, Santini F, Shaker JL, Sempos C, Ulivieri FM, Virtanen JK, Napoli N, Schafer AL, Bilezikian JP (2023) Vitamin D status and supplementation before and after Bariatric Surgery: Recommendations based on a systematic review and meta-analysis. Rev Endocr Metab Disord. 10.1007/s11154-023-09831-3 37665480 10.1007/s11154-023-09831-3 PMC10698146

[25] Inge TH, Courcoulas AP, Jenkins TM, Michalsky MP, Helmrath MA, Brandt ML, Harmon CM, Zeller MH, Chen MK, Xanthakos SA, Horlick M, Buncher CR (2016) Weight loss and health status 3 years after bariatric surgery in adolescents. N Engl J Med 374(2):113–123 26544725 10.1056/NEJMoa1506699 PMC4810437

[26] Al-Mutawa A, Al-Sabah S, Anderson AK, Al-Mutawa M (2018) Evaluation of nutritional status post laparoscopic sleeve gastrectomy-5-year outcomes. Obes Surg 28(6):1473–1483 29197046 10.1007/s11695-017-3041-7

[27] Peterson LA, Cheskin LJ, Furtado M, Papas K, Schweitzer MA, Magnuson TH, Steele KE (2016) Malnutrition in bariatric surgery candidates: multiple micronutrient deficiencies prior to surgery. Obes Surg 26(4):833–838 26297429 10.1007/s11695-015-1844-y

[28] van Rutte PW, Aarts EO, Smulders JF, Nienhuijs SW (2014) Nutrient deficiencies before and after sleeve gastrectomy. Obes Surg 24(10):1639–1646 24706197 10.1007/s11695-014-1225-y

[29] Aasheim ET, Hofsø D, Hjelmesaeth J, Birkeland KI, Bøhmer T (2008) Vitamin status in morbidly obese patients: a cross-sectional study. Am J Clin Nutr 87(2):362–369 18258626 10.1093/ajcn/87.2.362

[30] Parrott J, Frank L, Rabena R, Craggs-Dino L, Isom KA, Greiman L (2017) American society for metabolic and bariatric surgery integrated health nutritional guidelines for the surgical weight loss patient 2016 update: micronutrients. Surg Obes Relat Dis 13(5):727–741 28392254 10.1016/j.soard.2016.12.018

[31] Sandvik J, Bjerkan KK, Græslie H, Hoff DAL, Johnsen G, Klöckner C, Mårvik R, Nymo S, Hyldmo ÅA, Kulseng BE (2021) Iron deficiency and anemia 10 years after Roux-en-Y gastric bypass for severe obesity. Front Endocrinol (Lausanne) 12:679066 34630319 10.3389/fendo.2021.679066 PMC8493084

[32] Bjørklund G, Peana M, Pivina L, Dosa A, Aaseth J, Semenova Y, Chirumbolo S, Medici S, Dadar M, Costea DO (2021) Iron deficiency in obesity and after bariatric surgery. Biomolecules 11(5):613 33918997 10.3390/biom11050613 PMC8142987

[33] Mechanick JI, Apovian C, Brethauer S, Garvey WT, Joffe AM, Kim J, Kushner RF, Lindquist R, Pessah-Pollack R, Seger J, Urman RD, Adams S, Cleek JB, Correa R, Figaro MK, Flanders K, Grams J, Hurley DL, Kothari S, Seger MV, Still CD (2020) Clinical practice guidelines for the perioperative nutrition, metabolic, and nonsurgical support of patients undergoing bariatric procedures—2019 update: cosponsored by American Association of Clinical Endocrinologists/American College of Endocrinology, The Obesity Society, American Society for Metabolic & Bariatric Surgery, Obesity Medicine Association, and American Society of Anesthesiologists. Surg Obes Relat Dis 16(2):175–247 31917200 10.1016/j.soard.2019.10.025

[34] Guéant JL, Guéant-Rodriguez RM, Alpers DH (2022) Vitamin B12 absorption and malabsorption. Vitam Horm 119:241–274 35337622 10.1016/bs.vh.2022.01.016

[35] Al Mansoori A, Shakoor H, Ali HI, Feehan J, Al Dhaheri AS, Cheikh Ismail L, Bosevski M, Apostolopoulos V, Stojanovska L (2021) The effects of bariatric surgery on vitamin b status and mental health. Nutrients 13(4):1383 33923999 10.3390/nu13041383 PMC8073305

[36] Kwon Y, Ha J, Lee YH, Kim D, Lee CM, Kim JH, Park S (2022) Comparative risk of anemia and related micronutrient deficiencies after Roux-en-Y gastric bypass and sleeve gastrectomy in patients with obesity: an updated meta-analysis of randomized controlled trials. Obes Rev 23(4):e13419 35048495 10.1111/obr.13419

[37] Antoniewicz A, Kalinowski P, Kotulecka KJ, Kocoń P, Paluszkiewicz R, Remiszewski P, Zieniewicz K (2019) Nutritional deficiencies in patients after Roux-en-Y gastric bypass and sleeve gastrectomy during 12-month follow-up. Obes Surg 29(10):3277–3284 31201694 10.1007/s11695-019-03985-3

[38] Institute of Medicine Standing Committee on the Scientific Evaluation of Dietary Reference I, its Panel on Folate OBV, Choline. The National Academies Collection: Reports funded by National Institutes of Health. Dietary Reference Intakes for Thiamin, Riboflavin, Niacin, Vitamin B(6), Folate, Vitamin B(12), Pantothenic Acid, Biotin, and Choline, 10.17226/6015. Washington (DC): National Academies Press (US) Copyright © 1998, National Academy of Sciences; 1998.

[39] Khan KM, Jialal I. Folic acid deficiency. StatPearls. Treasure Island (FL): StatPearls Publishing Copyright©2023, StatPearls Publishing LLC; 2023.

[40] Lupoli R, Lembo E, Saldalamacchia G, Avola CK, Angrisani L, Capaldo B (2017) Bariatric surgery and long-term nutritional issues. World J Diabetes 8(11):464–474 29204255 10.4239/wjd.v8.i11.464 PMC5700383

[41] Paccou J, Caiazzo R, Lespessailles E, Cortet B (2022) Bariatric surgery and osteoporosis. Calcif Tissue Int 110(5):576–591 33403429 10.1007/s00223-020-00798-w

[42] Schafer AL (2017) Vitamin D and intestinal calcium transport after bariatric surgery. J Steroid Biochem Mol Biol 173:202–210 28027914 10.1016/j.jsbmb.2016.12.012 PMC5483209

[43] Yu EW, Kim SC, Sturgeon DJ, Lindeman KG, Weissman JS (2019) Fracture risk after Roux-en-Y gastric bypass vs adjustable gastric banding among medicare beneficiaries. JAMA Surg 154(8):746–753 31090893 10.1001/jamasurg.2019.1157 PMC6537834

[44] Ahlin S, Peltonen M, Sjöholm K, Anveden Å, Jacobson P, Andersson-Assarsson JC, Taube M, Larsson I, Lohmander LS, Näslund I, Svensson PA, Carlsson LMS (2020) Fracture risk after three bariatric surgery procedures in Swedish obese subjects: up to 26 years follow-up of a controlled intervention study. J Intern Med 287(5):546–557 32128923 10.1111/joim.13020

[45] Ablett AD, Boyle BR, Avenell A (2019) Fractures in adults after weight loss from bariatric surgery and weight management programs for obesity: systematic review and meta-analysis. Obes Surg 29(4):1327–1342 30725431 10.1007/s11695-018-03685-4

[46] Maghrabi AH, Wolski K, Abood B, Licata A, Pothier C, Bhatt DL, Nissen S, Brethauer SA, Kirwan JP, Schauer PR, Kashyap SR (2015) Two-year outcomes on bone density and fracture incidence in patients with T2DM randomized to bariatric surgery versus intensive medical therapy. Obesity (Silver Spring) 23(12):2344–2348 26193177 10.1002/oby.21150 PMC4701611

[47] Hofsø D, Nordstrand N, Johnson LK, Karlsen TI, Hager H, Jenssen T, Bollerslev J, Godang K, Sandbu R, Røislien J, Hjelmesaeth J (2010) Obesity-related cardiovascular risk factors after weight loss: a clinical trial comparing gastric bypass surgery and intensive lifestyle intervention. Eur J Endocrinol 163(5):735–745 20798226 10.1530/EJE-10-0514 PMC2950661

[48] Courcoulas AP, Goodpaster BH, Eagleton JK, Belle SH, Kalarchian MA, Lang W, Toledo FG, Jakicic JM (2014) Surgical vs medical treatments for type 2 diabetes mellitus: a randomized clinical trial. JAMA Surg 149(7):707–715 24899268 10.1001/jamasurg.2014.467 PMC4106661

[49] Buscemi S, Buscemi C, Corleo D, De Pergola G, Caldarella R, Meli F, Randazzo C, Milazzo S, Barile AM, Rosafio G, Settipani V, Gurrera S, Borzì AM, Ciaccio M (2021) Obesity and circulating levels of vitamin d before and after weight loss induced by a very low-calorie ketogenic diet. Nutrients 13(6):1829 34071985 10.3390/nu13061829 PMC8226843

[50] Wiley KD, Gupta M. Vitamin B1 (thiamine) deficiency. StatPearls. Treasure Island (FL): StatPearls Publishing Copyright© 2023, StatPearls Publishing LLC; 2023. 30725889

[51] Lange J, Königsrainer A (2019) Malnutrition as a complication of bariatric surgery—a clear and present danger? Visc Med 35(5):305–311 31768394 10.1159/000503040 PMC6873028

[52] Stroh C, Meyer F, Manger T (2014) Beriberi, a severe complication after metabolic surgery - review of the literature. Obes Facts 7(4):246–252 25095897 10.1159/000366012 PMC5644786

[53] Martel JL, Kerndt CC, Doshi H, Franklin DS. Vitamin B1 (thiamine). StatPearls. Treasure Island (FL): StatPearls Publishing Copyright© 2023, StatPearls Publishing LLC; 2023. 29493982

[54] Institute of Medicine Panel on M. Dietary Reference Intakes for Vitamin A, Vitamin K, Arsenic, Boron, Chromium, Copper, Iodine, Iron, Manganese, Molybdenum, Nickel, Silicon, Vanadium, and Zinc, 10.17226/10026. Washington (DC): National Academies Press (US) Copyright 2001 by the National Academy of Sciences. All rights reserved; 2001. 25057538

[55] Alvarez-Leite JI (2004) Nutrient deficiencies secondary to bariatric surgery. Curr Opin Clin Nutr Metab Care 7(5):569–575 15295278 10.1097/00075197-200409000-00010

[56] Moizé V, Andreu A, Flores L, Torres F, Ibarzabal A, Delgado S, Lacy A, Rodriguez L, Vidal J (2013) Long-term dietary intake and nutritional deficiencies following sleeve gastrectomy or Roux-En-Y gastric bypass in a mediterranean population. J Acad Nutr Diet 113(3):400–410 23438491 10.1016/j.jand.2012.11.013

[57] Smelt HJM, Pouwels S, Smulders JF, Hazebroek EJ (2020) Patient adherence to multivitamin supplementation after bariatric surgery: a narrative review. J Nutr Sci 9:e46 33101663 10.1017/jns.2020.41 PMC7550964

[58] Neri B, Bigarelli P, Filippi F, Belligoli A, Bettini S, Busetto L, di Cola RS, Giardiello C, Paolini B, Del Ciondolo I (2020) Efficacy evaluation of a medical food supplementation as prevention and therapy of nutritional deficiency in bariatric surgery. IJMDAT 3:e235

